# Root Endophytism by *Pochonia chlamydosporia* Affects Defense-Gene Expression in Leaves of Monocot and Dicot Hosts under Multiple Biotic Interactions

**DOI:** 10.3390/plants10040718

**Published:** 2021-04-07

**Authors:** Shimaa R. T. Tolba, Laura C. Rosso, Isabella Pentimone, Mariantonietta Colagiero, Mahmoud M. A. Moustafa, Ibrahim I. S. Elshawaf, Giovanni Bubici, Maria Isabella Prigigallo, Aurelio Ciancio

**Affiliations:** 1 Department of Genetics and Genetic Engineering, Faculty of Agriculture, Benha University, Moshtohor 13736, Egypt; shimaa.rashad@fagr.bu.edu.eg (S.R.T.T.); mahmoud.mustafa@fagr.bu.edu.eg (M.M.A.M.); i.elshawaf@fagr.bu.edu.eg (I.I.S.E.); 2 Istituto per la Protezione Sostenibile delle Piante, Consiglio Nazionale delle Ricerche, via G. Amendola 122/D, 70126 Bari, Italy; isabella.pentimone@ipsp.cnr.it (I.P.); mariantonietta.colagiero@ipsp.cnr.it (M.C.); giovanni.bubici@ipsp.cnr (G.B.); mariaisabella.prigigallo@ipsp.cnr.it (M.I.P.); aurelio.ciancio@ipsp.cnr.it (A.C.)

**Keywords:** *Fusarium oxysporum*, gene expression, lipoxygenase, *Meloidogyne incognita*, *Musa acuminata*, phenylalanine ammonia lyase, *Phytophthora infestans*, plant defense, Pratylenchus goodeyi, *Solanum lycoperiscum*

## Abstract

A study was carried out on the effect of the root endophytic fungus *Pochonia chlamydosporia* on plant systemic signal of defense related genes during fungal or nematode parasitism. Different biotic stress factors were examined, inoculating roots of dicot and monocot hosts with the endophyte, and measuring the expression of defense genes in leaves. A first greenhouse assay was carried out on expression of *PAL*, *PIN II*, *PR1* and *LOX D* in leaves of tomato cv Tondino inoculated with *Phytophthora infestans* (CBS 120920), inoculating or not the roots of infected plants with *P. chlamydosporia* DSM 26985. In a second assay, plants of banana (*Musa acuminata* cv Grand Naine) were artificially infected with *Fusarium oxysporum* f. sp. *cubense* Tropical race 4 (TR4) and inoculated or not with DSM 26985. In a further experiment, banana plants were inoculated or not with *P. chlamydosporia* plus juveniles of the root knot nematode (RKN) *Meloidogyne incognita*. A similar assay was also carried out in vitro with adults and juveniles of the lesion nematode *Pratylenchus goodeyi*. Differential expression of the defense genes examined was observed for all plant-stress associations, indicative of early, upward systemic signals induced by the endophyte. Changes in expression profiles included a 5-fold down-regulation of *PIN II* at 2 dai in leaves of tomato plants treated with *P. infestans* and/or *P. chlamydosporia*, and the up-regulation of *PAL* by the endophyte alone, at 2 and 7 dai. In the TR4 assay, *PR1* was significantly up-regulated at 7 dai in banana leaves, but only in the *P. chlamydosporia* treated plants. At 10 dai, *PIN II* expression was significantly higher in leaves of plants inoculated only with TR4. The banana-RKN assay showed a *PR1* expression significantly higher than controls at 4 and 7 dai in plants inoculated with *P. chlamydosporia* alone, and a down-regulation at 4 dai in leaves of plants also inoculated with RKN, with a *PR1* differential up-regulation at 10 dai. *Pratylenchus goodeyi* down-regulated *PIN* at 21 dai, with or without the endophyte, as well as *PAL* but only in presence of *P. chlamydosporia*. When inoculated alone, the endophyte up-regulated *PR1* and *LOX*. The gene expression patterns observed in leaves suggest specific and time-dependent relationships linking host plants and *P. chlamydosporia* in presence of biotic stress factors, functional to a systemic, although complex, activation of defense genes.

## 1. Introduction

Available strategies for sustainable management of plant pathogens and pests include the use of antagonists such as biological control agents and/or endophytes. Among the arsenal of beneficial microorganisms present in the rhizosphere, a number of hyphomycetes have been investigated due to their complex mechanisms of action, including parasitism of invertebrate pests flanked by root endophytism. Examples of beneficial endophytic interactions, based on multiple traits, include species of *Trichoderma* and *Metarhizium*, non-pathogenic *Fusarium* spp., and the nematode egg parasite *Pochonia chlamydosporia* (Goddard) Zare & Gams [1,2,3,4]. The evolutionary, selective adaptations and mechanisms underpinning this dual behavior—endophytism and invertebrate parasitism—are not yet fully elucidated.

Such an alliance between beneficial endophytes and plants, active in rhizosphere tri-trophic interactions, represents a useful evolutive trait of these microorganisms, with potential in crop management. Several mechanisms characterizing endophytic fungi are indicative of a mutual and beneficial interaction with the host plants. They include direct antibiosis and mycoparasitism, as well as induced resistance and hormone signaling [2]. Endophytes can sustain plant growth by eliciting or priming their defense responses, often systemically [5,6]. Endophyte colonization can activate defense responses at the molecular level, upon additional biotic or abiotic stresses including root-associated microorganisms or pathogens, with an increase of the defense-related gene expression [6,7]. In the case of *P. chlamydosporia*, roots of monocot and dicot host plants showed that the endophyte colonization induced the expression of several defense genes in roots, eliciting an early host defense response, which is effective in the presence of pests such as endoparasitic nematodes [8,9,10,11]. Endophytic *P. chlamydosporia* induced changes in the transcriptome of tomato roots, highlighting a specific modulation of stress-responsive transcripts related to a selective activation of defense pathways [11]. The endophyte affected the root expression of key genes of the jasmonic acid (JA) pathway, such as lipoxygenases. This pathway has also been associated with systemically induced defense against nematode invasion [12,13], suggesting a beneficial effect of *P. chlamydosporia*. Also, groups of well-known salicylic acid (SA)-responsive genes, such as phenylalanine ammonia lyase (*PAL*) were clearly affected by the fungus 21 days after infection (dai) [11]. Interestingly, the tomato genes not expressed in the presence of *P. chlamydosporia*, at the early stage of interaction, included several glutaredoxins (GRXs). It has been recently shown that GRXs regulate the activity of basic leucine zipper-type transcription factors which interact with NPR1 and are essential for the regulation of many SA-responsive genes, such as *PR1* [14].

Considering the complexity of the pathways and interactions involved, several facets of the biochemical factors involved in the plant–endophyte and pathogen interplay are, however, still unknown, and worth further investigation. Few data are available on the signals or effectors triggered, on the plant tissues involved, or on the genes differentially expressed when the endophyte coexists inside the plant with one or more pathogens. The effects exerted on above-ground plant parts are also unknown, in particular when endophytes such as *P. chlamydosporia* are applied to roots or soil [6]. Therefore, the objective of this study was to evaluate the effect of *P. chlamydosporia* on the expression of defense-response-related genes when colonizing host plants exposed to different pathogens and parasites. After inoculating roots with *P. chlamydosporia* and exposing plants to different sources of biotic stress, we monitored the expression of the following defense-related genes: proteinase inhibitor II (*PIN II*), phenylalanine ammonia-lyase (*PAL*), lipoxygenase (*LOX*), and pathogenesis-related protein 1 (*PR1*). These genes were selected because, in a previous study, they were found to be differentially expressed in tomato roots colonized by *P. chlamydosporia* [11]. Their analysis was later extended to banana to check if they were consistently elicited in monocots too. The host and pathogens/pest associations studied were tomato (*Solanum lycoperiscum* L.) inoculated with *Phytophthora infestans* (Mont.) de Bary, banana (*Musa acuminata* L.) challenged with *Fusarium oxysporum* f. sp. *cubense*(E.F. Smith) Snyder & Hansen, Tropical Race 4 (Foc TR4, the causal agent of Fusarium wilt), the root-knot nematode *Meloidogyne incognita* Kofoid & White (Chitw.) (RKN), and the lesion nematode *Pratylenchus goodeyi* Sher & Allen.

## 2. Results

Data from the different assays supported the hypothesis that root endophytism by *P. chlamydosporia* elicited a defense response in the leaves of the inoculated plants tested, in the presence or absence of a biotic stress factor. The expression levels of the gene examined, however, varied with the different stress sources applied and the time of sampling. The statistical significance observed in the different assays for the comparisons among all treatments are shown in Supplementary Tables S1–S4. The endophytic colonization by *P. chlamydosporia* was confirmed in inoculated roots, with or without *Phytophthora infestans, Pratylenchus goodeyi,* or RKN, by qPCR assays. No significant difference was observed in colonization among treatments, except for *Phytophthora infestans* assay (Supplementary Figure S1).

### 2.1. Effect of P. chlamydosporia and P. infestans on Tomato

The tomato plants inoculated with *P. infestans* CBS 120190 already showed minor leaf discolorations and damages 2 days after inoculation (dai) (Supplementary Figure S2A). The presence of *P. chlamydosporia* in the root tissues of the inoculated plants was confirmed at 7 dai by PCR amplification of a fragment of the fungus VCP1 (alcaline serinprotease, GenBank AJ427460)-encoding gene. The mean VCP1 amplicon amounts were the highest in the plants inoculated only with *P. chlamydosporia*, compared with the *P. infestans*-parasitized plants (Supplementary Figure S1). The analyses of the mRNAs of the selected defense genes were carried out from leaves for the quantitative determination of the corresponding transcripts. The PCR primers yielded single products of the expected lengths for the target genes (*PINII, PAL*, *LOX D,* and *PR1*) and the actin control (Table 1).

**Figure 1:**
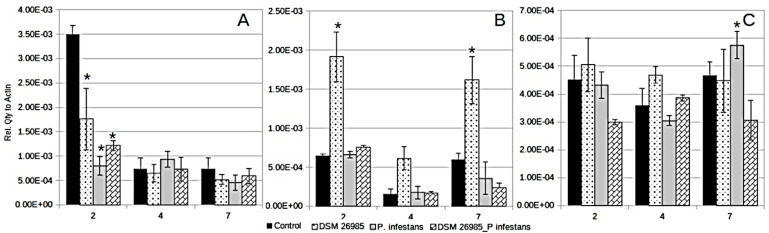
Gene expression of proteinase inhibitor II (*PIN II*) (**A**), phenylalanine ammonia lyase (*PAL*) (**B**), and pathogenesis-related protein 1 (*PR1*) (**C**) in leaves of *Solanum lycopersicum* plants at 2, 4, and 7 days after infection (dai) with Phytophthora *infestans* and/or Pochonia *chlamydosporia* DSM 26985. Asterisks show significant differences from the untreated control at each dai, as shown by Least Significant Difference (LSD) test (* *p* < 0.05; bars = SE).

All treatments with fungal infections showed a reduced expression of *PIN II* at 2 dai, with a higher effect observed in the treatments including *P. infestans* (Figure 1A). The *PIN II* expression did not show significant differences among treatments at 4 and 7 dai (Figure 1A). *PAL* expression appeared differentially up-regulated, compared to the control, in the treatment with *P. chlamydosporia* alone, which was significantly higher when compared to all other treatments at 2, 4, and 7 dai (Figure 1B). Although decreased at 4 dai, *PAL* expression in the *P. chlamydosporia*-alone treatment was significantly higher when compared to the *P. chlamydosporia* plus *P. infestans* treatments at 2, 4, and 7 dai (Figure 1B).

No significant difference with respect to the control was observed in the expression of *PR1* at 2 and 4 dai. At 2 dai plants inoculated only with *P. chlamydosporia* showed a significantly higher expression (*p* < 0.05) than in the *P. chlamydosporia* plus *P. infestans* inoculation (Figure 1C). *PR1* expression was significantly different from the control at 7 dai, showing the highest expression in the *P. infestans* treatment and a down-regulation in presence of both fungi (Figure 1C). The expression of the lipoxygenase D gene could only be detected at 7 dai, however, it was not significantly different (data not show).

### 2.2. Effect of P. chlamydosporia and Foc on Banana

**Figure 2:**
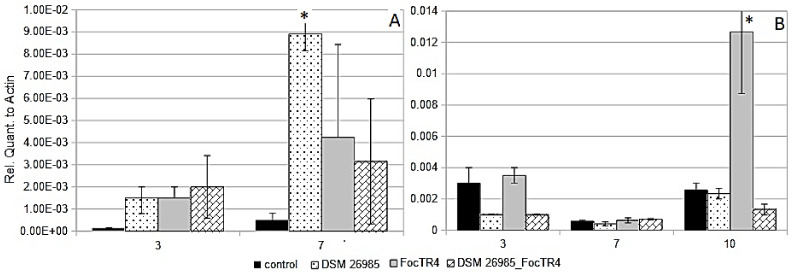
*PR1* (**A**) and *PIN II* (**B**) expression in leaves of banana cv Gran Enana plants at 3, 7, and 10 dai inoculated with FocTR4 isolate NRRL36114 (CBS 102025) and/or *P. chlamydosporia* DSM 26985. Asterisks show significant difference from the untreated control (* *p* < 0.05; bars = SE).

Although no visible symptoms were observed in *Fusarium oxysporum* f. sp. *cubense* (FocTR4)-inoculated Gran Enana plants at 10 dai, Foc TR4 was re-isolated from soil and surface sterilized roots. A previous assay showed that the fungus induced severe damage in the inoculated plants, which died at 60 dai, an effect that was only partially mitigated in the *P. chlamydosporia*-inoculated plants (Supplementary Figure S3). Actin-normalized gene expression data showed a systemic response with over-expression of *PR1* in leaves of inoculated banana plants at 3 and 7 dai, significantly higher than the control at 7 dai, only for the plants treated with *P. chlamydosporia* alone (Figure 2A). Infection by FocTR4 reduced *PR1* expression at 7 dai in the plants inoculated with *P. chlamydosporia* (Figure 2A).

At 10 dai *PIN II* expression was significantly higher in leaves of plants inoculated only with FocTR4, with respect to the control (Figure 2B). *PAL* expression at 7 dai did not show significant differences among treatments or in comparison with the control. No expression of *LOX* was found in the experimental condition tested.

### 2.3. Effect of P. chlamydosporia and M. incognita on Gene Expression in Banana Leaves

**Figure 3:**
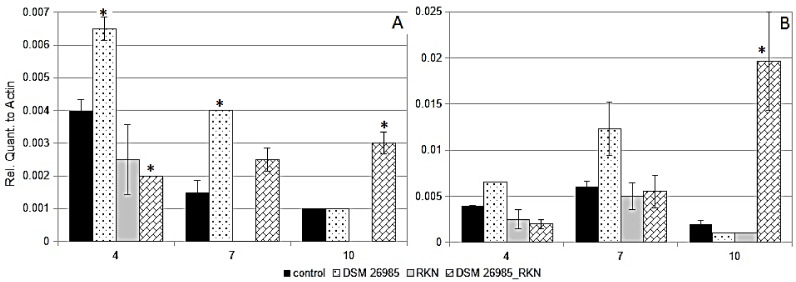
*PR1* (**A**) and *PIN II* (**B**) expression in leaves of Gran Enana plants at 4, 7, and 10 dai inoculated with RKN *M. incognita* L4 and/or *P. chlamydosporia* isolate DSM 26985. Asterisks show significant difference from each dai untreated control, as shown by LSD test (* *p* < 0.05; bars = SE).

The banana–RKN-inoculated plants showed the symptoms of root infestation as indicated by the root galls, that were already induced by the nematode at 10 dai (Supplementary Figure S2C,D). Leaves of plants inoculated with *P. chlamydosporia* alone showed a *PR1* expression significantly higher than the corresponding controls at 4 and 7 dai (Figure 3A). 

No difference from the control was found at 10 dai, when transcript amounts decreased reaching a level similar to the control (Figure 3A). The plants doubly inoculated (RKN and *P. chlamydosporia*) showed an opposite trend, with significantly lower amounts of *PR1* transcripts at 4 dai, with a significant increase of expression at 10 dai (Figure 3A). *PR1* expression was lower than the control at 4 dai in leaves of plants inoculated only with RKN, and could not be detected in the following sampling times (Figure 3A). A significant increase of *PIN II* expression was observed in doubly inoculated plants after 10 days of RKN infection, contrasting the trend observed in untreated parasitized plants (Figure 3B). No significant differential expression was observed among the other treatments. *PAL* expression was observed in leaves of plants with *P. chlamydosporia* inoculation and/or parasitized by RKN but no significant change was found among treatments and the control, except for the plants inoculated with RKN only at 4 dai (data not show). *LOX* expression was observed at 4 dai for RKN infection only (data not show).

### 2.4. Effect of P. chlamydosporia and P. goodeyi on Gene Expression in Banana Leaves

**Figure 4:**
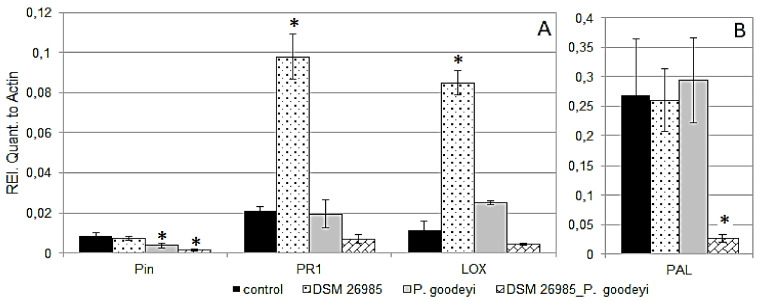
Gene expression of *PIN II*, *PR1*, lipoxygenase (*LOX*) (**A**) and *PAL* (**B**) at 21 dai in leaves of Gran Enana plants inoculated with *P. goodeyi* and/or *P. chlamydosporia* DSM 26985. Asterisks show significant differences from each untreated control, as shown by LSD test (* *p* < 0.05; bars = SE).

At 21 dai the plants inoculated in vitro with *P. goodeyi* showed root necrotic areas induced by the nematode attacks. Endophytic colonization by *P. chlamydosporia* was confirmed in all fungus-inoculated plants. Differing from the RKN-inoculated plants, the mean amount of VCP1 amplicons in the *P. goodeyi*-infested roots was lower than in the roots inoculated only with the endophyte, although not statistically different (Supplementary Figure S1). Significant changes were observed in the expression of defense genes in leaves at this time, related to the nematode and/or fungus inoculations. *PR1* and *LOX* showed a significantly higher expression only in leaves of plants treated with *P. chlamydosporia*, whereas *PIN II* was reduced in the plants treated with *P. goodeyi* with or without *P. chlamydosporia* inoculation (Figure 4A). A significant down-regulation was also observed for *PAL* at 21 dai, however limited only to the leaves of doubly inoculated plants (Figure 4B).

## 3. Discussion

Several experimental data showed that beneficial microorganism from the root microbiome can affect plant health. A variety of root-associated mutualistic fungi, including *Trichoderma* spp. and mycorrhizae, were capable of sensitizing the host plant immune system, enhancing a defense response [11,15,16]. In banana, a bioprotective effect of AM fungi was reported compared to migratory endoparasitic nematodes such as *Radopholus similis*, *Pratylenchus coffeae* and *P. goodeyi* [17,18,19]. Therefore, microorganisms able to induce a systemic resistance appear as an important soil biological resource. However, the mechanism activated to prime the whole plant for enhanced defense is only partially elucidated. Previous studies reported that *P. chlamydosporia* colonization may underpin a defense response in roots [9,10,11]. The assays carried out with different pathogens and plant hosts confirmed the capacity of *P. chlamydosporia* to promote a systemically induced response before or after the insurgence of a biotic stress factor. However, it may vary in relation to the pest/pathogen and the genes examined.

Roots colonized by *P. chlamydosporia* differentially modulated the expression of key genes involved in plant defense responses in leaves of both monocot and dicot hosts. Moreover, the endophyte is known to colonize roots without inducing significant damage [10,20]. The genes herein examined (*PIN II, PAL, LOX*, and *PR1*) are involved in defense pathways by encoding pathogenesis-related proteins or JA- or SA-signaling molecules. In both *S. lycopersicum* and *M. acuminata* the interaction with *P. chlamydosporia* showed a down-regulation of *PIN II* suggesting that endophytism modulated its expression. *PIN II* plays an important role in tomato defense from abiotic and biotic stress factors [15,21,22]. Most of plant proteinase inhibitors (PI) act on pathogens by interacting with the active sites of their protease targets [23,24,25], thereby forming a stable inhibitory complex. In *P. infestans*-treated plants, the almost 5-fold down-regulation of *PIN II* occurred in leaves as early as 2 dai (Figure 1A), suggesting that neutralization of the host proteinase inhibitors is achieved by the pathogen during the early infection phase.

During progression of the *P. infestans* infection, proteases are known to accumulate around haustoria in host tissues, thus preventing the host defensive reaction [26]. Banana plants inoculated with *Pochonia chlamydosporia* showed a similar response in the interaction with the highly pathogenic Foc TR4. The various formae specialis of *F. oxysporum* are generally regarded as hemi-biotrophs, where the initial infection occurs as a biotroph, later switching to a necrotroph as the plant defense system reacts to the biotrophic invasion [27,28]. Our investigation confirms these observations. In fact, significant up-regulation of *PIN II* was observed only at 10 dai and not at the earliest phase of interaction (3 and 7 dai) (Figure 2B).

Endophytism by *P. chlamydosporia* differentially modulated *PIN II* expression in banana plants under nematode interactions, while during double biotic stress a high increment at 10 dai was observed in the *M. incognita*–fungus system and for *P. goodeyi* an inhibition occurred at this sampling time. These results suggest that the response associated to the protease and PI effectors secreted during the interaction is specific to the invading pest. The different host response could also be caused by the nematode feeding behavior, as *P. goodeyi* is a migratory endoparasite causing lesions, whereas in case of the sedentary RKN endoparasitism no injury occurs, apart from local histological changes. It is therefore possible that a number of biochemical signals occur during the host and pathogen/parasite early molecular interplay. Biochemical mechanisms leading to the adaptation and counter-adaptation between pathogens and their host plants have been recognized in many host–pathogens interactions, including RNA interference and effector proteins [29,30].

The levels of *PIN II* may therefore be useful in testing host responses to pathogens in selective assays, as its expression is associated with a healthy condition based on an active defense system. It is worth to note that a similar *PIN II* down-regulation was observed for the *P. chlamydosporia*-treated plants, although only to a minor extent. Data suggest that *PIN II* down-regulation is also required for root penetration, but that the differences between the colonization outcomes, and damage induction, between *P. infestans* and *P. chlamydosporia* likely depend on differences in host-response mechanisms and/or affected defense pathways.

*PAL*-encoded phenylalanine ammonia–lyase is involved in the initiation of the polypropanoid biosynthesis pathway trough L-phenylalanine conversion to *trans*-cinnamate, leading to the production of several secondary metabolites. In tomato *PAL* is involved in plant development and defense [31,32,33]. Its transcript was almost three-fold differentially up-regulated only in the *P. chlamydosporia*-treated control, at all sampling times, suggesting an enduring involvement of this gene during roots endophytic colonization. In banana leaves, *PAL* expression appeared to be unaffected by the *P. chlamydosporia* colonization. However, a differential systemic signal was observed during the double stress tested. Double biotic stress resulted in a down-regulation of *PAL* in leaves of plants inoculated with *P. goodeyi* and *P. chlamydosporia*, in comparison to plants inoculated only with *P. chlamydosporia* or the control. Previous studies in banana germplasm showed that *PAL* was strongly induced in resistant plants and down-regulated (or not induced) in susceptible germplasm, during the early biotrophic phase of infection by the pathogenic fungus *Mycosphaerella fijiensis* [34]. Considering that resistant germplasm is not yet available for *P. goodeyi* and Foc 4, our results confirm that *PAL* down-regulation is involved in the susceptibility of banana cv Gran Enana to these pathogens.

Differences in *LOX D* expression were only detected in tomato leaves at 7 dai, when the transcripts were down-regulated in either *P. chlamydosporia-* or *P. infestans*-treated plants. However, the two inocula together did not differ from the untreated control. *LOX* expression were only detected in banana leaves at 21 dai with a marked up-regulation with respect to uninoculated plants. Interestingly, during double interaction with *P. goodeyi*, *LOX* expression dropped drastically. Lipoxygenases are involved in the wound-induced JA pathway, activating a local and a systemically-induced defense, thereby increasing resistance to *P. infestans* [21,22,23]. Although inhibition of *LOX D* by *P. infestans* and *P. chlamydosporia* alone may be ascribed, respectively, to leaf tissues and root colonization, its expression in presence of both inocula suggests the occurrence of a contrasting mechanism, likely induced by the endophyte [11].

Finally, in spite of its reported role in the inhibition of *P. infestans* development in leaves [35], *PR1* did not show significant changes among treatments and times, suggesting either a reduced functionality of this gene in the tested tomato line, or a host sensitivity targeting infections by other pathogens. Instead, studies conducted on resistant banana genotypes during interaction with *M. fijiensis* showed an increased expression of genes coding for the pathogenesis-related (PR) proteins [34,35,36], suggesting that the pathogen-associated molecular-pattern-triggered immunity and the effector-triggered immunity responses, observed in other monocots and dicots, are conserved in *Musa* spp. [37]. Our data showed that the interaction of *M. acuminata* with *P. chlamydosporia* produced an up-regulation of *PR1* at early (Figure 2A and Figure 3A) and later stages (Figure 4A). These patterns suggest that, during endophytism, *P. chlamydosporia* can modulate the plant *PR1* expression. A reduction in *PR1* levels was observed, however, during the double interaction with Foc TR4, suggesting that the pathogen can also inhibit the response triggered by *P. chlamydosporia*. On the other hand, under nematode interaction, while *PR1* expression in RNK-inoculated plants decreased reaching undetectable levels after 7 days, plants inoculated with *P. chlamydosporia* and RKN maintained the expression of *PR1*, up to 10 days. When comparing the different stress sources, *P. chlamydosporia* alone up-regulated *PR1* at 7 dai, either in TR4- or RKN-treated banana plants, and at 21 dai in those with *P. goodeyi*. However, *PR1* expression showed two opposite trends in time, with a progressive decrease for *P. chlamydosporia* alone, and an increasing trend in plants inoculated with the endophyte and stressed by either RKN or TR4.

In this study we demonstrate that *P. chlamydosporia* endophytism produced differential expression of defense genes, eliciting or inhibiting their leaf activity in an early defense response, both in monocot and dicot hosts. It is worth to note that *P. chlamydosporia* conidial inocula were added to soil, and that the fungus effects were observed on leaves, likely suggesting the occurrence of one or more direct/indirect, upward biochemical signals.

## 4. Materials and Methods

Leaves of artificially inoculated monocot and dicot hosts were analyzed to identify possible common factors when investigating the above-ground response in plants.

### 4.1. P. chlamydosporia and P. infestans Assay on Tomato

Tomato cv Tondino seeds were sterilized on the surface (2.5% hypochlorite) and germinated on 1.5% water agar at 25 °C in the dark. Subsequently, seedlings were transferred into 15-cm diameter pots containing 0.8 L of a mixture of soil and sand (1:1) sterilized by autoclaving (120 °C, 1 atm for 20 min). Assays were carried out in a greenhouse at 24–26 °C when plants reached 4-weeks old. The *P. chlamydosporia* isolate DSM 26985 present in collection at the CNR Institute for Sustainable Plant Protection (IPSP, Bari, Italy), was used as endophyte. Inoculation was carried out by adding, per pot and around the seedling, 1 mL of a conidial suspension with 1.8 × 10^6^ propagules × mL^−1^. *Phytophthora infestans* CBS 120920, was multiplied on pea agar at 20 °C and used for the greenhouse assay. Inoculation was performed in three points onto an apical leaf area using a 20 μL suspension contained around 640 propagules (hyphal fragments and sporangia) obtained by scraping the surface of a growing colony in sterile distilled water (SDW). The experimental design included four treatments—an untreated control, soil inoculation with DSM 26985 only, leaf inoculation of *P. infestans* with previous soil inoculation with DSM 26985, and inoculation with *P. infestans* only. Leaf samples were taken in triplicates at intervals of 2, 4, and 7 dai with *P. infestans*. Infection was checked by observing the presence of symptoms on treated leaves.

### 4.2. P. chlamydosporia and Foc Assay on Banana

Single banana cv Gran Enana plants proceeding from an in vitro culture (Cultesa, Tenerife, Spain) were transplanted in plastic containers with turf. Subsequently, at height around 8–15 cm, the plants were transplanted in 2 L pots with autoclaved soil (50% sand, 50% loamy soil) and kept in a greenhouse at 24–25 ± 4 °C. *Fusarium oxysporum* f. sp. *cubense* NRRL36114 (CBS 102025), isolated from Pisang Manurung in Indonesia, was acquired from Westerdijk Fungal Biodiversity Institute NL (authorization MiPAAF n. 31519, dated 06/12/2017). *Pochonia chlamydosporia* (DSM 26985) was inoculated 3 days before TR4 inoculation, directly on soil by pipetting in three holes around roots 7 × 10^6^ propagules per plant. Inoculation was carried out by mycelium adding three 1-cm-wide agar blocks per pot. The plants were maintained in a quarantine greenhouse under controlled conditions at 24–26 °C and regularly watered. The experimental design included four treatments—an untreated control, soil inoculation with DSM 26985 only, soil inoculation with FocTR4 plus subsequent soil inoculation with DSM 26985, and inoculation with FocTR4 only. The samples (around 200 mg of tissue) were collected from apical leaves in four replicates for subsequent gene expression analysis. Sampling times were 3, 7, and 10 dai with FocTR4. At end point an aliquot of soil and pieces of surface sterilized roots were spread on water agar plates to check for *P. chlamydosporia* and FocTR4 presence.

### 4.3. Pochonia chlamydosporia and RKN Assay on Banana

Plants of banana cv Gran Enana maintained in a greenhouse, as previously described, were inoculated with 1 mL of a conidial suspension containing 1.8 × 10^6^ conidia × mL^−1^ of *P. chlamydosporia* DSM 26985, as previously described. After 4 days, the pots were inoculated with 2000 juveniles (J2) and approximately 1000 vital eggs of the *M. incognita* of population MILEV-L4, originating from a single egg mass and proceeding from Leverano (Italy) and multiplied in a greenhouse on cherry tomato [38]. The infective individuals (J2 larvae) were obtained from eggs extracted from the infested roots by the hypochlorite method [39,40], and allowed to hatch in tap water at room temperature (20–24 °C), under a continuous air flow produced by a submersed peristaltic pump outlet. After inoculation, plants were maintained in greenhouse at 24–25 ± 4 °C with regular irrigation. The experimental design included four treatments—an untreated control, application of DSM 26985 only, inoculation with RKN only, and inoculation with DSM 26985 plus RKN, with four replicates each. The samples (around 200 mg of tissue) were collected from the banana apical leaves. Sampling time were 4, 7, and 10 dai with RKN. At the end-point, the plants were explanted and galling symptoms were checked under a light microscope.

### 4.4. Pochonia chlamydosporia and P. goodeyi Assay on Banana

The assay was carried out using in vitro plants of Gran Enana (ITC1256) obtained from Biodiversity and maintained on Murashige medium in a growth chamber (Sanyo Electric MLR-351, Osaka, Japan) at 26 °C with 16 h illumination. At height of around 8–12 cm, plants were transferred into 100 mL Magenta boxes contained sterile vermiculite:sand (1:1) and inoculated with 5 × 10^5^ conidia of *P. chlamydosporia* DSM 26985 per plant, as previously described. After 4 days, the pots were inoculated with 300 specimens of *P. goodeyi.* The nematodes were extracted from banana soil and root fragments proceeding from samples collected by Coplaca (Cooperativas Bananeras, Sta Cruz de Tenerife, Canary Islands, Spain) in a naturally infested farm in Tenerife. Single specimens were hand-picked from the tap water suspension, washed in several baths of sterile tap water (STW) in watch glasses, surface sterilized by a 10-min immersion in an antibiotic solution and then inoculated in a few STW droplets directly on the banana roots. Plants were kept for 21 days in a growth chamber as previously described. The experimental design included four treatments—an untreated control, application of DSM 26985 only, inoculation with *P. goodeyi* only, and inoculation with DSM 26985 and *P. goodeyi*. After 21 days the samples (around 100 mg tissues) were collected from the apical leaves in four replicates. At end point, plants were explanted and roots observed under a light microscope.

### 4.5. Real-Time PCR and Gene Expression Analyses

Leaf tissues were powdered in liquid nitrogen to extract RNA using a TRIzol™ Reagent solution (Invitrogen) or a Rneasy Mini Kit (Qiagen). Retrotranscription was performed for almost 500 ng of RNA using Superscript IV (Invitrogen). qPCR analyses were carried out in an Aria (Agilent) or Mx3000P (Stratagene) device, using SYBR green master mix (AllGene or Promega) and specific primers. 

Primers were designed in order to amplify the following genes: phenylalanine ammonia-lyase (*PAL*), pathogenesis-related protein (*PR1*), lipoxygenase (*LOX*), and proteinase inhibitor II (*PINII*). Available sequences of *S. lycopersicum* SL4.0 (https://solgenomics.net/organism/Solanum_lycopersicum/genome/ (accessed on 11 March 2021) and *M. acuminata* (DH-Pahang_pseudochromosomes_2.0 (https://banana-genomehub.southgreen.fr (accessed on 24 February 2021) matching the enzymes of interest were checked by blastx (https://blast.ncbi.nlm.nih.gov/, accessed on 11 March 2021). After confirming the presence of conserved domains for each protein, primers were selected using Primer3 and BLAST tool (https://www.ncbi.nlm.nih.gov/tools/primer-blast/ (accessed on 15 September 2017 for Tomato and 12 December 2018 for Banana) (Supplementary Table S5). Primers were tested for specificity and efficiency and the best-performing were used for qPCR (Table 1). Gene expression was calculated using AriaMx version 1.6 or Mx3000P version 3.2 software and the relative quantitation expressed-like mRNA target gene quantity with respect to a normalizer gene (actin) was used for comparison.

### 4.6. Analysis of P. chlamydosporia Endophytic Colonization by qPCR

Plant roots were collected at the last time-point assay and conserved at −80 °C. Total DNA was extracted from around 100 mg of tissue using a plant/fungi DNA isolation kit (Norgen Biotek) following the manufacturer’s instructions. Total DNA was quantified by nanodrop and a 50 ng/µL concentration solution was prepared for amplification reactions. For *P. chlamydosporia* detection and quantification, qPCR experiments were conducted in an Aria (Agilent Scientific Instruments, Santa Clara, CA, USA) PCR device with VCP1 as the target gene, using the primers For0, (5′-ctcgaggctgcccaac) and Rev0 (5′-tgcatgcactaggctcgg) [41]. Amplification reactions were performed in a 15 µL volume with 2 x SYBR green master mix (AllGene), 50 ng of total DNA and 500 nM of primer. The thermal profile was 95 °C for 3min, 40 cycles at 95 °C for 30 s, 52 °C for 20 s, and 72 °C for 10 s. A standard curve was constructed, using serial dilutions from 1 ng to 1 pg of genomic DNA of *P. chlamydosporia*. The initial amount of fungal DNA contained in total root DNA was calculated by correlation of quantification cycle (Cq) values with Cq values in the standard curve. Data significance were evaluated by applying Student’s t-test (*p* ≤ 0.05).

## Figures and Tables

**Table 1:** Primers used for qPCR amplification and amplicon details.

	Primer Pair	Genomic Location	Amplicon Efficiency		
			Length	Tm (°C)	%
*Solanum lycopersicum* Genome Version SL4.0	PAL f-gacagcaggaaggaatccaa	SL4.0ch00:1898125..1898106	158	82.2	82
	PAL r-caaccaaatagggattcgaca	SL4.0ch00:1897969..1897989			
	PR1 f-tgccaagaccggtggtaatttc	SL4.0ch09:691132..691153	101	86	83
	PR1 r-tgcccgctagcacattggt	SL4.0ch09:691233..691215			
	PINII f-ttgttgtgcaggcagtaagg	SL4.0ch11:13068648..13068667	152	80.5	77
	PINII r-ggctcacgcgtaattattgaa	SL4.0ch11:13068800..13068780			
	LOXD f-ttggcaccaagttcaggccc	SL4.0ch03:64660076..64660095	231	79	73
	LOXD r-tggacttaagctagtattag	SL4.0ch03:64660307..64660288			
	ACTfast f-aggcaggatttgctggtgatgatgct	SL4.0ch03:45371071..45371096	106	83.7	85
	ACTfast r-tacgcatccttctgtcccattccga	SL4.0ch03:45371176..45371152			
*Musa acuminata* DH-Pahang Pseudochromosomes 2.0	PAL f-ctggactacgggttcaaggg	Chr01:2940377..2940396	93	82.2	82
	PAL r-ctggacgtggttggtaacgg	Chr01: 2940469..2940450			
	PR1 f-tacgcctacggggagaacat	Chr04:2013348..2013468	86	86	83
	PR1 r-tgcttctcctccacccactt	Chr04: 2013433..2013414			
	PINII f-agtacatgacctgcaactcc	Chr04:4858280..4858300	113	80.5	77
	PINII r-ctgcagtttacctccattgc	Chr04: 4858392..4858372			
	LOX6 f-tatcaacacactccccagat	Chr06:31522043..31522063	173	81.5	81
	LOX6 r-cgctcctgttcttcagataa	Chr06:31521870..31522050			
	ACTfast f-aggcaggatttgctggtgatgatgct	Chr02:24048458..24048433	106	83.9	85
	ACTfast r-tacgcatccttctgtcccattccga	Chr02:24048353..24048377			

## Data Availability

The data presented in this study are available within the article and its supplementary material).

## Supplementary Materials

The following are available online at https://www.mdpi.com/article/10.3390/plants10040718/s1, Figure S1: Root endophytic colonization by *Pochonia chlamydosporia*, Figure S2: Symptoms induced by inoculation of tomato leaf with *Phytophthora infestans* and on roots of banana after inoculation with *Meloidogyne incognita*, Figure S3: Symptom severity of Fusarium wilt in pot-grown banana plants, Tables S1–S4: Statistical comparison for gene expression in tomato and banana plants, Table S5: Procedure adopted for primers design.
